# It’s ‘*probably the teacher!*’ A strategic framework for clinical staff engagement in clinical student bullying intervention

**DOI:** 10.1186/s12909-019-1552-8

**Published:** 2019-04-25

**Authors:** Althea Gamble Blakey, Kelby Smith-Han, Lynley Anderson, Emma Collins, Elizabeth K. Berryman, Tim Wilkinson

**Affiliations:** 1 Otago School of Medicine, University of Otago, Dunedin, NZ New Zealand; 2 Department, Bioethics Centre, University of Otago, 71 Frederick St, PO Box 56, Dunedin, 9054 New Zealand; 3 Otago Polytechnic and Staff Nurse, Southern District Health Board, Dunedin, NZ New Zealand; 4 North Shore Hospital, Waitemata District Health Board, Auckland, New Zealand

**Keywords:** Bullying, Mistreatment, Intervention, Clinical environment, Engagement, Staff development

## Abstract

**Background:**

Student bullying in clinical practice persists, and poor outcomes continue: for learning, academic achievement and career goals, for their mental and physical health and potentially affecting all staff and patients in a clinical workplace. We describe an emergent framework for the strategic design of a bullying intervention, presented as a staff development opportunity.

**Methods:**

CAPLE (Creating A Positive Learning Environment) was a bullying intervention designed around current best evidence about ameliorating student bullying in the clinical environment. CAPLE was also an action research project delivered in two eight- week cycles, one in 2016 & another in 2017. CAPLE’s primary practical foci were to offer clinical staff in two separate hospital wards an opportunity to develop their clinical teaching skills and to guide them in reflection and cultivation of values around students and learning. Research foci were: 1. to gain insight into staff experiences of CAPLE as a development process and 2. to evaluate how CAPLE might best help staff reflect on, discuss and develop values around student learning, to include bullying. Staff undertook five active learning workshops combined with supportive contact with one researcher over the research period. Data include individual interviews, staff and researchers’ reflective journals and a paper survey about staff experiences of the 2017 intervention.

**Results:**

We confirm the effectiveness of best evidence from the literature and also that a strategic four-part framework of *approach, process, content* and *person* can further enhance a bullying intervention by increasing the likelihood of participant engagement, learning and values change.

**Conclusions:**

This research aggregates and adds weight to the current literature about student bullying and adds important pragmatic detail about best practice for bullying intervention design and delivery. Ultimately, this emergent framework offers insight to help move past some persistent barriers encountered by those wishing to improve workplace behaviour.

## Introduction

*… the tools provided to us … have the power to change an age old culture embedded in blame and inequality. I have had to examine my own practice and ensure that I adopt an attitude that reflects the behaviours that I expect from my colleagues* ([1], p. 47).

Student bullying in the clinical workplace is a global problem, largely without a known, effective solution [2–4]. Despite being significantly under-reported [3, 4] student bullying still has an exceptionally high prevalence, described in an extensive literature across the healthcare sector. One indicator of this prevalence is a recent review of the literature, which indicates that an average of 59% of medical students experience bullying during their clinical training [4].

The nature of clinical student bullying is also well described. The commonest reported bullying acts have been found to be verbal and physical harassment, gender and racial discrimination and, importantly, several forms of academic harassment [3–5]. While any student can suffer bullying, at the hands of any staff member [6–8], students of minority ethnicity, or sexuality, and of the female gender^1^ are likely to experience it more [4]. A bullying perpetrator can be any staff member, but have been found to most likely be senior staff members [3, 4].

Because of its potential severity and persistence, student bullying can substantially influence the student *and* the performance of the health service in which it takes place. Bullying can negatively affect a student’s lifelong learning [9], clinical and academic performance [4], physical and mental health, and career opportunities [10–12], but also negatively affect the ongoing functioning of all staff in a workplace [2, 3]. The latter can be to the extent that avoidable adverse outcomes and rates of medical error increase [3, 4].

### Defining bullying

While there is currently no widely accepted definition of what constitutes clinical student bullying, we define bullying/mistreatment as explicated by Mavis ([13], p.706). This definition appropriately acknowledges the student’s potentially vulnerable position in the workforce, and one that sees a percentage prevalence of bullying almost twice that of senior staff ([13], p. 706):

Mistreatment, either intentional or unintentional occurs when behaviour shows disrespect for the dignity of others and unreasonably interferes with the learning process. Examples of mistreatment include sexual harassment; discrimination or harassment based on race, religion, ethnicity, gender, or sexual orientation; humiliation; psychological or physical punishment; and the use of grading and other forms of assessment in a punitive manner.

### Detailing the problem

In the academic health sector, bullying interventions continue to be trialled and in some cases, researched and reported - see Swiggart [14]. Despite the literature being generally extensive, such as that about the consequences of bullying, there is sparse and piecemeal pragmatic detail available with which to plan an intervention to help students. In other words, it is difficult to know what exactly to do to effectively engage staff in learning about student bullying, and moving them on to improve their behaviour [15].

In some cases, lack of detail in the literature might be explained by bullying interventions undertaken without a research component; the information has simply not been described, evaluated or disseminated. However, because of the various methods of administration and evaluation reported in the literature that *is* available, there still seems an apparent lack of aggregated, detailed guidance around which to design an effective intervention.

From the literature, however, these is other evidence emerging which seems helpful because it indicates why some interventions might be ineffective. One example is that an intervention approached in a way that appears to ‘target’ a specific staff group or unwanted behaviour can be less effective than that which includes all staff and has a positive focus; participants report these foci to represent implicit criticism of their behaviour, or themselves as a person. Specifically, some interventions have been shown to fail because percieved criticism can engender feelings of inadequacy, which can ultimately lead to defensiveness, failure to engage and learn from an intervention, or worse, active protest or resistance to it [3, 15, 16].

### Defining engagement

Participants’ reports about engagement in learning are the main phenomena by which we judge the effectiveness of each CAPLE process we report here. While a detailed discussion of engagement in learning is outside the scope of this paper, for reference, we indicate that we take a psychological perspective to understanding engagement in learning. This perspective, summarised by Kahu [17], allows several potential influences on engagement to be considered, and that we draw on as part of this paper. These include a learner’s behaviour, the behaviour of others and psychosocial processes relating to emotions such as fear.

### Research aims and questions

As can be usual with action research, research questions can be proposed at its outset, arise as part of a broader study question or aim, or develop singularly during the research process itself [18]. Here, we broadly aimed to put together and evaluate an anti-bullying intervention developed from fragmented evidence about ameliorating student bullying. A related question then emerged as part of this broader question: ‘*what helped staff engagement in CAPLE?*

## Methods

To guide those wishing to replicate our study and research, we describe the development and administration of the CAPLE intervention as well as its associated research methods.

### The CAPLE projects

The CAPLE (Creating a Positive Learning Environment) projects were strategic anti-bullying interventions developed in response to reports of persistent worldwide student bullying and reports of concerning local levels at the hospital in question [19]. The CAPLE interventions utilised best available evidence around ‘what works and what doesn’t work’ to ameliorate student bullying. Each CAPLE was a bullying intervention offered to staff at a different clinical department in an Australasian teaching hospital and respectively administered in 2016 & 2017. Each CAPLE project entailed a unique approach of workshops about developing staffs’ clinical teaching skills, and working and supporting them to reflect on and develop related values. As indicated, at its outset, CAPLE was primarily informed by a literature review, and the results from 2016 used to inform the 2017 project. There are 4 more cycles of CAPLE currently being planned or in progress.

We worked with the following conclusions from the literature about ameliorating student bullying. In summary, a more useful approach to ameliorating student bullying engages staff by:

1. incorporating an understanding of potential workplace bullying catalysts, such as burnout [20] and personal values [3] which, when ignored, can perpetuate bullying [21–23];
2. considering staffs’ unique work context [24–27] and addressing their adult learning needs [15, 28, 29] rather than ‘lecturing’ them [29];
3. operating over and above policy/reporting about bullying behaviour which are necessary, but not sufficient processes for ensuring behaviour change [3, 30–34];
4. including all staff, without targeting specific groups [15, 16];
5. focusing on cultivating skills (e.g. clinical teaching) rather than seeking to punitively eliminate negative behaviour [1, 35–38];
6. being administered by a skilful interventionist, understood as credible and engaging, without taking a top-down approach [1, 15].

The CAPLE intervention was then created and implemented. The broader CAPLE team comprised six highly experienced clinicians, educators and researchers. The ‘front line’ workshops and contact with staff were administered by Althea Gamble Blakey (AGB) and Kelby Smith-Han (KSH).

In each of the two 2016 & 2017 projects, extensive negotiation with hospital and clinical service management enabled a clinical site to be identified and accessed, and CAPLE introduced to managerial staff at the site via email. This site was selected on the understanding that bullying was likely to exist in most departments, rather than a focus on what was actually happening there. Both sites were acute intervention, short stay clinical sites, and staff therefore worked in a highly pressured environment. Questions and queries from potential participants were used as opportunities for AGB and KSH to begin to establish functional and trusting relationships with each. With the support of senior staff, six doctors and six nurses of varying levels of experience were recruited as key participants.

CAPLE workshops were designed around the emergent conclusions from the literature. Workshops employed active learning methods, and each was a 25 min session held local to the participants’ workplace. Each covered one topic about clinical teaching and learning per week (total six), selected broadly around the idea that a positive focus which aimed to develop skills would be more effective than some others, as explained by Thomas [37] and Thompson [38]. We also selected topics, and the level of delivery, based on what seemed to be current challenges for, and needs of participants, described in initial individual interviews. This approach was thus a context-specific and ‘bottom-up.’ Topics included ‘teaching under time pressure,’ ‘giving effective feedback’ and ‘fear in learning.’ If a CAPLE key participant was unable to attend an arranged workshop, they were offered it again, at their convenience. Thus, each topic was workshopped several times a week, with varying size groups (1–25 staff). All staff at each site were invited to attend the workshops, as well as formal participants in the broader research.

The researchers who delivered CAPLE, which included workshop facilitation, were selected for their extensive expertise in small group teaching, clinical teaching/learning, developing values, and for being the ‘kind of person’ able to help staff engage in learning, determined by recent evaluations of their teaching. These measures indicated the researchers to be caring, trustworthy, respectful and approachable, all qualities and values noted to be helpful for, if not crucial to learner engagement, especially the adult learner [38–40].

Each key participant was assigned a specific researcher to undertake their entry and exit interviews and stay in touch throughout the 8- week research period. Contact was casual, by email or in person, as requested. One example of such contact was that AGB sent a brief ‘how’s it going?’ email once a week, to ask how participants were finding the application of workshop material in practice, and chatted further/met with participants as required, for example, if they described difficulties with a teaching method, or had strong feelings as a result of their teaching which they wanted to address or investigate.

### Research methods and data collection

CAPLE was embedded within an action research methodology, considered to be eminently suitable to the development and refinement of solutions to problems or questions about practice [20–24], here, about teaching clinical staff who are potentially bullying their students. Specifically, we aimed to iteratively identify issues of concern and to cultivate, test and evaluate solutions [25, 26]. This paper represents data collected in the first 2 cycles of CAPLE.

CAPLE began with an interview (20–40 min) of each key participant, about their experiences of clinical teaching and learning, including experiences with bullying, if raised. Our semi-structured technique allowed scope to widen conversation to emergent issues specific to each workplace and person. Workshops were then given, and participants stayed in contact with the researcher throughout this time. Interviews were repeated after the workshop series, with a specific focus on what worked to help participants engage in learning, and why.

Having undertaken analysis of the 2016 data, small adjustments were made to CAPLE processes and methods for the 2017 study. This kind of adjustment to research methods is usual in action research and can be one way to increase research quality and reliability [18]. Importantly, the workshops were refined in terms of what was felt, and said by participants to ‘work’ for staff in 2016 (timing, topic focus etc.). A short ‘exit’ survey was also instigated for 2017 participants. The survey was given in person and returned by internal mail to the researcher less well known to the participants (e.g. AGB received KSH’s participant surveys). With this, we sought triangulation between specific findings of each study and wanted participants to be free from worry that they might offend the researcher if they wrote negative comments. We also realised the importance of the exit interview in obtaining evaluative comments about the researchers as workshop facilitators and support persons. Thus, we also added another short post-interview, done by another researcher. Again, we did this to minimise potential participant bias towards the researcher and ensure we gathered honest and useful feedback which may have been negative.

Data thus comprise contributions from 24 participants & two researchers, across two action research projects, addressing similar issues about teaching and learning, and, as it arose, student (and staff) bullying. Specifically, data include:

- individual interview transcripts
- emails between participants and researchers
- field notes made about interactions between researcher and participants
- reflective journals in which all participants (to include researchers, as customary with action research) recorded thoughts and experiences about their teaching practices and CAPLE project processes
- a short ‘exit’ survey administered to participants (2017 study), which included general questions, about participants’ judgements of CAPLE workshop teaching and three specific questions about teacher values (respect, integrity, caring) identified from 2016 study data. Questions were mostly evaluated with a Likert scale of ‘Yes, Possibily, Unsure, Possibly not, No’ with space open for further comment. The comments form the basis of what is reported here about the survey.

### Data analysis

We analysed spoken and freehand data with an general inductive approach [41], creating themes from emerging ideas to accurately represent our meaning. Our ideas and themes were thus developed in line with a constructivist epistemology [42, 43]. In essence, we sought answers to research questions with an open mind as to what data might reveal.

AGB, KSH and LA (Lynley Anderson) undertook the data analysis, creating themes around issues that arose, and adding/removing themes as discussions continued, in pursuit of ways to accurately categorise and explain the findings. Analysis finished when no more themes emerged and no data remained unclassified [43]. Throughout the process, themes and raw data were taken to the wider author group to check for accuracy of meaning and thematic classification.

### Data representation

We represent data variously: in our own words to summarise discussion between staff participant groups; verbatim quotations from interviews, emails between staff and researchers and participants’ reflective journals. Where wording is changed to preserve confidentiality, meaning was preserved by checking with the participant concerned.

## Results

We describe emergent evidence in themes developed from the analysis of both CAPLE project data. Between these projects and data collection methods we found substantial triangulated data about how to engage participants in reflecting on, learning about and in developing clinical teaching skills and related values, as part of addressing student bullying. We report data from the specific perspectives of key participants and researchers, in response to the question: *What helped staff engagement in CAPLE?*

In summary, this research confirmed the current best evidence to be effective but also revealed further specific details about how to best engage clinical staff in learning about clinical teaching and student bullying. We begin with a brief illustration of findings that allowed us to judge CAPLE was successful in engaging participants.

### What did we want to see? Evidencing effective engagement

The primary, broad aims of most staff development activities are to aid learning and its transfer to the workplace [44]. In the case of CAPLE, we aimed to help staff: 1. learn/refine clinical teaching skills, 2. learn to reflect on teaching practice, behaviours to students and underlying values and as a result 3. implement enhanced skills and improved behaviours in the workplace.

We take participants’ reports that they had implemented what they had learned on CAPLE into the workplace as a strong indication that they had effectively engaged in CAPLE, as well as first-hand reports about specific features they found engaging. Here, a doctor describes his developing teaching skills – we use such quotes as representative of several other similar quotes from data:

*I had a great fortnight with a TI* [Trainee Intern, 6th year medical student]*, which is unprecedented. I felt I had loads more ways to teach up my sleeve and the confidence to put it into action*.

(Doctor 1 (2016), Reflective Journal)

Here, Doctor 1 reflects on an earlier student interaction:

*… I ended up talking to* [a student] *like a father would to his children. It was not good … unfortunately I went in a bit headstrong. I’m not going to do that again. But, at least, in reflection I know what I’ve done and I will try not to do it again!*

(Doctor 1, discussion with AGB)

Here, Doctor 1 reflects on his changing behaviour:

*… another doctor said to me ‘this* [mistake] *never happened to me while I was on* [another ward]*. I think it’s all your fault.’ I said back to him that is was inappropriate to say that at the time. I was quite frankly disgusted … I wouldn’t have done that* [spoken up] *before* [before CAPLE].

(Doctor 1, discussion with AGB)

Four major themes emerged from data, here entitled: approach, process, content and person, which we present by explaining why we chose to implement these specific features of CAPLE, and offer evidence from participants about how these features helped them engage.

### Theme 1 approach: avoiding targeting specific staff, and any staff

CAPLE was approached as a positive, multidisciplinary project with a focus on clinical teaching. We developed this approach on an understanding that failure to engage participants in learning has been found to result from an intervention that ‘targets’ a staff group or negative behaviour [3, 15, 16]. For example, aiming to recruit only ‘nurses’ might result in resentment and failure to engage this staff group, or appearing to target ‘communication’ could alienate or offend.

Doctor 1 described the elements of CAPLE’s approach which were helpful to his engagement, by comparison with that of another staff development opportunity, apparently implemented with an approach aimed at ‘Improving Communication’ for the medical staff. He interpreted the approach to this intervention thus:

*I felt like a naughty kid being told off...we were just send to it* [the course] *and told to do stuff, no explanation or reason, and just not very nice. They clearly didn’t give a damn about how it all feels. I didn’t get into it and I certainly don’t use the stuff* [what was taught].

Similarly, comments from Nurse 5 (2017):

*They* [management] *just put the* [other] *program in the hospital, it’s like we’re being told off.*

Confirming CAPLE to be more effective in approach, Nurse 6 (2017):

[CAPLE] *empowers you to say ‘no’ and be in control … it* [the CAPLE approach] *implies that you think we are OK* [we are good people] *and going well...Responding to our context, but not in a patronising or offensive way.*

Having established that our approach was effective for engaging participants in learning, we discussed other results that we classified as ‘approach,’ but were different. We found that some of CAPLE’s results about ‘approach’ emanated from necessary pre-research interactions with senior management, in other words, they were about engagement of staff outside of our formal participant group.

*I heard they don’t want us to do the project, because they don’t want to be associated with a bullying project. It* [the inference of bullying] *means they have a problem... Even if they are doing something about the bullying. We are going to have to tread* ***very*** *carefully … .. I think I’m going to have to stop making references to ‘bullying.’*

(AGB, written reflection on verbal report to research group, 2016)

We therefore found that the best *approach* to CAPLE needed to address and minimise potential for CAPLE to lead to *any* staff feeling targeted, including line management in the wider hospital setting. Not only could ‘targeting’ affect participant engagement in an intervention itself, but also staffs’ engagement with our researchers’ discussions, recruitment of potential participants but could also lead to a refusal to host the intervention at all.

We thus added the following to our ‘no targeting’ approach to CAPLE, to include further and better ways to approach *all* staff for effective engagement. To -

- avoid reference to ‘bullying’ verbally and in documentation, unless raised by others
- to reassure management that we were coming to their department to use their knowledge, rather than because we had been informed of an issue with student bullying
- use ‘circumspect’ language to discuss bullying, e.g. euphemisms like ‘tricky behaviour’
- present CAPLE as research, with an accompanying emphasis on optional participation and that participants would be contributing to a specific cause
- present CAPLE as an opportunity for the researchers to learn about staff development from participants, in particular in specific clinical work contexts that can be challenging

### Theme 2 process: using active learning processes with participant support

#### Active learning

CAPLE’s aims were to help clinical staff develop teaching skills and reflect on practice and related values. We understood these aims to require pedagogic processes congruent with participant engagement in such topics; we need to teach them as they were best learned. We looked to the literature on education, as well as bullying, and instigated proven pedagogic method and process:

- workshops as a host to active learning processes appropriate for adult learners [39, 44, 45]
- small groups, congruent with engagement in discussion [39] and reflection [44], and about developing skills of teaching [39] and sensitive topics such as bullying and personal values [39, 40, 46–48]
- teachers with substantial expertise in facilitating adult small group learning [39, 47].

Evidence that participants found these pedagogic processes helpful for engagement was plentiful. Again, described in contrast with another staff development opportunity given at this work site:

… *we weren’t talked at or bored, that really turns me off.* (Nurse 6, 2017)

*… nothing good will come of staff sitting in a lecture about this stuff and being told stuff.* (Doctor 1, 2016)

*… such a different approach than a PowerPoint. It was great and really got me thinking.* (Doctor 8, 2017)

In contrast, CAPLE participants indicated that experiences of workshops were more positive:

… *therapeutic … they give you chance to think and then have the skill to bring all your thinking together … good to get things off my chest, especially to talk it over and reflect with someone who has a different perspective and come to a new conclusion.* (Doctor 8, 2017)

*… slightly free-form element which can be a little worrying* [at first] … *but by the end we realised we had actually covered quite a lot of stuff, so it’s good. You’ve been taken through stuff without realising that you have been taken through it! And it’s a great place to talk through tricky things, without feeling embarrassed. I felt relaxed and engaged through the whole thing.* (Doctor 7, 2017)

### Participant support for positive values change

We offered participants personal support during CAPLE, guided by our understanding that some learning would necessarily concern values or discussion of difficult experiences. As either topic can be eminently sensitive, we understood some participants might benefit from ongoing support to ‘get through’ the thinking which might necessarily happen afterwards, in a potentially ongoing period of values development. We understood that ongoing engagement and achievement of some learning aims would depend on some staff being provided with ongoing support [30, 48, 49].

A specific example of support helping participants to stay engaged in values change was offered in a discussion with Doctor 2 about offering effective feedback to a clinical student who they disliked. In summary, Doctor 2 reported that his engagement in discussing, reflecting and finding ways to deal with this issue was dependant on the ‘*safe space’* created by the researcher; that his thinking processes would have otherwise somehow become ‘*distracted and waylaid’* because it was ‘*too hard thinking about why I don’t like him* [the student], *but I know I need to*.’

Further examples about support helping participant engagement in learning about teaching, and in values change emerged from CAPLE 2017 exit survey and interviews. We asked participants specifically what it was about CAPLE that helped them engage and learn:

*… it legitimises the way I feel and think, being able to talk to someone about my teaching … One-on-one chats were great, we made a great connection.* (Nurse 7, 2017)

*The one-on-one discussions give me a chance to think about things myself rather than in the group, where I might just sit back.* (Doctor 7, 2017)

*The one-to-one mentoring … to learn I need to talk rather than listen or write. It’s even better to talk to someone who can help and give me support with that.* (Doctor 8, 2017)

### Theme 3 content - relevant, useful and legitimate

CAPLE’s initial content focus aimed to help develop participants’ clinical teaching skills, guided by an understanding that such a focus can help improve things for students and improve general workplace culture. For example, learning to give feedback well can help students learn and staff to communicate [50] and a lack of teaching skill can, for some, catalyse student bullying [50, 51] say, out of frustration.

Our topic selection was initially confirmed as being relevant and useful by participant entry interview data and more latterly the 2017 exit survey, in which participants indicated that this hospital’s professional support for clinical teaching skill development was comparatively and universally lacking.

#### Make content wholly relevant and useful to everyone

Participants indicated that they engaged in CAPLE because it was relevant to their work, both in specific content and the level at which it was pitched:

*… going back to the beginning of teaching stuff was SO useful. I really got into it.* (Doctor 5, 2017)

*I’m genuinely disappointed it’s over* [CAPLE], *it’s not just therapeutic, its more than that, it’s reminded me of stuff I have forgotten and to remind me why I do what I do. I feel better at teaching now and I hope that I am better too.* (Nurse 7, 2017 exit interview)

In her reflective journal, AGB offered further reasoning why CAPLE topic helped staff engage: that workshops were relevant, but also not superfluous which might have been the case, say, if a workshop on communication skills was offered, but staff already had well developed communication skills.

#### Legitimacy

We found that *legitimacy* was also important to staff engagement in CAPLE: a participants’ general sense that a topic (here, bullying) was somehow acceptable for discussion, not ‘off limits.’ Via workshops’ general focus on clinical teaching, we were able to include bullying as part of discussion because of the natural relationship between bullying and teaching, such as academic bullying [5, 36] or how one might behave to a student that you dislike. References to bullying thus came across to participants as incidental, part of a wider or related discussion, perhaps experienced as a ‘softened blow’ in comparison with direct references, which can sound accusatory:

*… it’s a good way to do it because conversations can be about mostly something else, and it doesn’t sound as accusatory as having it as the only thing.* (AGB, journal, 2017)

*… it’s like it’s somehow legitimised, isn’t it, if you go at it ‘from the side’ as it were. It’s not as confrontational, is it, as the other way which goes at it hammer and tongs* [colloquialism for ‘rather brutal’]*.* (AGB conversation with KSH, reported in reflective journal)

CAPLE participants ultimately seemed happy to accept references to bullying made within the remit of clinical teaching, one even remarking about the potential difficulties in framing a bullying intervention in ‘*another way.’*

*… I like how you presented it. You have changed the way I have thought about things which is important...but you have proven that this is a remit that allows us to talk about bullying, because as it’s about the fears and how you handle yourself and because some people would struggle doing it another way.* (Doctor 5, 2017)

### Theme 4 person – being the right person

CAPLE researchers were purposely selected for their expertise and being the ‘kind of people’ likely to work well in potentially challenging situations. Doctor 1, 2016, confirmed the importance of teacher skill to his own engagement and learning:

*… you were very patient, gave me a lot of time for reflection on my thoughts, pauses, time for me to explain, open to other ideas, very encouraging and skilful.* (Exit interview)

We also found that ‘being the right person’ - over and above skills and knowledge – to be confirmed as important to participant engagement. The ‘right person’ was understood as facets of ‘who the teacher was,’ revealed by Doctors 7 and 8 in our 2017 exit interviews with the second ‘unknown’ interviewer:

*… very comfortable interactions – non-threatening, engaging and empowering … the whole atmosphere of the workshop was mutually* ***respectful*** *, essential if you are to learn something … I felt I had learnt something about myself, too.*

*If you didn’t want to teach us* ***we would be able to tell*** *and that would not be good for learning.*

We asked participants about the teachers’ values specifically in the 2017 ‘triangulation’ exit survey:

“What was it specifically about the CAPLE teaching that worked, or not, for you?”

*Probably the teacher!*

We identified four specific values (the ‘right’ person) to be important to participant engagement: *respect, integrity, caring* and *Living that which you teach*

… *you could not show up, not keep appointments, be late, all those sorts of things, but instead you gave me things to read, you were prompt, you went over and above [what was necessary], I absolutely think you* ***care*** *about us and that* ***matters****. You sold a good project to me, but in the end I* ***trusted*** *you and that’s what matters.* (Doctor 7, 2017)

### Respect and engagement

Doctor 7*: From the first meeting I could tell that you had respect for us and what we do, and that it was mutual. I could tell by the way you spoke and handled yourself, and how you let us be experts in our field without it turning into a pissing contest* [‘trying to out-do’].

AGB: *What did it do for you, feeling that I respected you?*

Doctor 7: *It helped me engage in the project. And if I hadn’t have felt it, I wouldn’t have, even if I do already like to talk about teaching and learning.*

The researcher’s (AGB’s) journal throughout 2016 & 2017 offers further confirmation that the perception of respect could be helpful to participants engagemet in CAPLE:

*I had to work really hard to show them I respected them, even though some of what came out of their mouths made me want to not respect them. That man with the laptop for starters. They have to understand that I think they are* ***essentially good people*** *, otherwise it wont work, and they wont think about what they need to think about. Its bloody hard work!*

### Integrity and engagement

*Integrity* was described by a participant in comparison with different (non-CAPLE) workshop they had attended. He reported feeling that he ‘ … *just couldn’t believe’* the teacher of the workshop; that this person ‘*looked like they didn’t know what they were doing or even that they believe in it* [what they were teaching].’ ‘Integrity’ thus seems to embrace several ideas, such as *being qualified, credible*, *keen on teaching and believable.* Doctor 6 summarised:

*… I met with [the researcher] today...she’s very passionate about this project and teaching and clinical student learning, it’s invigorating that she shares this passion. She knows what she’s doing, too. I believe what she is saying, and it makes sense*. (Reflective Journal)

Nurse 8 and Doctor 7 reported that integrity (in its various sub-forms) helped them better engage with researchers and was important, and complementary to, *respect* (theme 1):

… that the researcher *‘looks like they want to be here and know what we do here*. *Those kinda go together, don’t they?’* (Nurse 8)

*… Their experience in teaching and learning is what makes them great to listen to, and not just some jumped-up qualification that can’t be applied in practice. They mean it all, too, they are doing it with good hearts and with our interests at heart too.* (Doctor 7)

### Caring and engagement

Participants also indicated that ‘caring’ was important to their engagement:

*I think you do care about us … to do a program like this you would struggle if you didn’t care, you’d feel, like, 'I’ve got to see those wankers today.'* (Doctor 5, 2017, exit interview)

One participant went as far as to say that a bully’s:

*… best chance* [to learn] *is probably how you are doing it, as you and they can then talk and get them reflecting on what they do … we’ve* [the staff] *had enough of that stuff that ‘targets’ bullies.*

### Living that which you teach

The final value reported as helpful for engagement could be described as more general than the others, perhaps encompassing several as yet unidentified individual values. Colloquially described as the researcher ‘living out’ the methods which they had themselves taught in the workshops, in their interactions with the participant. For example, that the researcher avoided making staff fearful:

[In discussion about a workshop on ‘Fear & Learning’] *I wouldn’t have been as impressed or* ***listened to what you said*** *if you hadn’t done exactly what you said* ***we*** *should do. I hadn’t thought about this before, but it’s* ***really*** *important.* (Nurse 7, 2017).

In illustration, Palmer ([60], p.4):

The question we most commonly ask [in teaching] is the ‘what’ question –what subjects shall we teach? When the conversation gets a bit deeper, we ask the ‘how’ question – what methods and techniques are required to teach well? Occasionally, when it goes deeper still, we ask the ‘why’ question – for what purpose and to what ends do we teach? But seldom, if ever, do we ask the ‘who’ question- who is the self that teaches? How does the quality of my selfhood form-or deform-the way I relate to my students, my subject, my colleagues, my world?

## Discussion

Student bullying in the clinical environment is persistent and costly in many senses, and we still need to understand how to better engage staff participants in interventions. We offer an emergent framework for the strategic development of a bullying intervention. We found bullying intervention *approach* to require careful engagement with *all* staff via a concomitant understanding of how to avoid ‘targeting.’ A focus on enhancing teaching and learning skills was palatable to staff; this focus on promoting desirable behaviours contrasts with what we suggest would be a less effective approach on removing undesirable behaviours. Pedagogic *process* should encourage active learning but also offer participants personal support, for optuimal engagement in long term thinking and values development. *Content* should be relevant and useful, given at an appropriate level and if possible, determined by participants and be such that discussion of bullying is legitimised and therefore acceptable to participants. The *person* undertaking an intervention needs to have specific skills and knowledge and exhibit positive values congruent with intended learning outcomes: ‘the right *person* for the job.’ To discuss findings, we reference appropriate literature and also describe what might eventuate if each emergent strategy is not employed as part of a bullying intervention.

### Approach

Implementing CAPLE required sound ethical principle and process. Staff working in the service that we wished to research were also fearful of attracting negative media attention. This concern may have heightened or exaggerated any pre-existing sensitivity about discussions relating to bullying in the workplace. However, one specific concern with CAPLE’s approach was that potential participants might be negatively influenced by other staff having concerns with it, a phenomenon which has been acknowledged in the literature [3, 45]. Thus, we feel that our overall positive, teaching and learning approach without direct reference to bullying was especially necessary. With this approach, we also felt able to engage well with the wider service staff *and* potential participants, to develop discussions about bullying as a related ‘side-issue,’ and maintain both our effectiveness and ethical transparency.

However, this approach to CAPLE was emotionally and cognitively demanding for the facilitator to maintain; avoiding direct reference to bullying was hard, as was dealing with rather defensive reactions from some staff (such as people looking at their laptops, rather than keeping eye contact with the facilitator). To cope with this, the facilitator took advantage of the considerable support offered by the wider CAPLE team, to ‘vent’ and discuss their reactions to staff, and thus sustain consistently helpful behaviour. We recommend those wishing to instigate a bullying intervention come to understand the importance of using such an approach but also to acknowledge that support should be an important, integral component to it.

Examining ‘approach’ from the point of view of ‘what if we don’t do it this way,’ we suggest that more direct references to bullying or to use an intervention that appears to target staff may result in:

- Reluctance to host, or attend an intervention (or research).
- Staff negatively influencing participants engagement.
- Potential participants feeling targetted and declining to take part.
- Participants attending workshops, but failing to engage in learning.

### Process

There were few surprises about the success of CAPLE workshop processes, as we understood that effective engagement in small group learning, and about sensitive topics, could be hard [47, 48, 52, 53] and that active learning methods can be crucial to engagement and learning [36]. However, participant support was, for some, necessary to achieve such goals. This finding makes sense, given that one ultimate aim of CAPLE was to change participant behaviour, an inherent challenge [54].

Reassuringly, the support processes offered as part of CAPLE were simple and effective, and appropriate for those working within a busy acute service with serious time constraints. While researcher availability was casual (by email, text) this finding is indicative of utility for a potentially moral issue: if a bullying intervention is likely to challenge personal values to encourage personal growth, such support could then be understood as imperative to success but also essential for the fulfilment of the employers duty of care towards its staff. In further illustration, we understand that failure to instigate active learning or participant support might result in:

- Participants feeling ‘talked at’/bored and failing to engage, potentially exascerbating bad behaviour [39, 40]
- Participants lacking sufficient guidance to support the application of skills/behaviour in the workplace and thus resulting in no positive behaviour change
- Participants initially engaging in discussion, but failing to accomplish the required reflection and thinking about values development, and resulting in no values change or positive behaviour change.

### Content

We understood a focus on teaching and learning as useful to engage participants in an anti-bullying intervention, on the basis that many aspects of general workplace culture can be attributed to, and depend on effective teaching and learning process [1, 39] and that teaching and learning is relevant to a substantial percentage of the workforce. This topic allowed us to legitimately raise bullying issues without the feared ‘targeting’ and potential resultant disengagement. A focus on teaching and learning also offered us an opportunity to influence staff behaviour in other contexts: having discussed some values, we saw newly cultivated values ‘crossing contexts’ [40, 47] here, to staff-staff interactions as well as staff–student. As such, we add to recent literature about values education and address a historical lacks of detail about on how exactly values education might be carried out in the classroom. Our contribution is that approaching such a difficult topic ‘carefully’ and somewhat laterally can help learning.

We also understand that failure to provide appropriate content, or containing‘direct’ content more directly about bullying might result in:

- Participants feeling targeted and failing to engage.
- Participants failing to learn skills appropriate to their learning, or workplace.
- Participants becoming disinterested and failing to engage or even to provide us with data about why this was so.

### Person

We understood that researchers’ skill and knowledge would likely influence participant engagement in CAPLE. We also had emerging evidence that a teacher’s values (‘who they were’) might do so too. The quote ‘*you guys weren’t arseholes, which was nice*,’ is a favourite exemplar of this phenomenon, which we consider to be *over and above* teachers’ skill or knowledge. While this comment might be understood as ‘faint praise,’ we also understood it as a paradoxical or negative framing of ‘what worked’ in the CAPLE project (see later), about the teacher as a person: participants confirmed this interpretation to indicate that our researchers had requisite values for participant engagement, while others (‘arseholes’) did not. While this data does not to connect the presence of a value and participant engagement, but rather supports a general finding that teacher values are important to learning, the value-engagement connection is understood from further exit interview data. In this data, the value of ‘respect’ was described by a participant as a direct correlate with their engagement.

The specific values reported here to be helpful for participant engagement are described in the teaching/staff development literature [55, 56], less so in that about bullying intervention. All three values (respect, integrity and caring) have been generally associated with the engenderment of positive emotion in the learner and, arguably, for some as essential for learning engagement [48, 56]. *Respect* is reported to positively support change [15]; *integrity* to help a learner entrust their learning to a teacher without fear, or shame [15]. *Caring* can have a potentially significant positive effect on engagement [50, 58] specifically via increased confidence [40, 46].

‘Living out what you teach’ might be initially understood as modelling in teaching; the researchers modelled to CAPLE participants what they wished participants to learn - see Swennen [59], which can significantly influence a learner [55–57] especially if sceptical or nervous [59]. However, the phenomenon we observed seemed ‘deeper’ than simply demonstrating a skill: participants specifically talked about ‘who teachers were’ affecting their engagement, here; for the teachers to be seen to value what they wanted the participants to learn to do. We provisionally interpret this phenomenon as one which connects teacher values [58] with the idea of congruent teaching [59].

Overall, these findings provide a strategic framework for bullying intervention that has utility in practice. We also understand several elements of the framework to have a moral imperative. There are moves (in NZ, 2015 [60]) to instigate legislation which states that institutions need to respond to bullying with methods which are *effective,* rather than just to *respond,* as part of their duty of care for each employee. If values and related behaviours are understood to be a focus of an intervention, and at the same time understood to offer participants emotional and cognitive challenges, the effectiveness of factors such as ongoing support could be understood as obligatory for success and mental health.

## Conclusion

This four-part strategic framework offers a sound basis for the development of a student bullying intervention, and aggregates and strengthens what we find in the literature. It offers pragmatic, nuanced ideas about what an intervention should entail to ensure better participant engagement, learning and results. As such, we feel our findings represent a potential turning point for student bullying research.

### Strengths and weaknesses of this research

Data collection methods were generally appropriate for our aims, but also had drawbacks. Some reports about staff reactions to CAPLE were by proxy and naturally subject to interpretation. However, because we used action research, we were afforded substantial opportunity to discuss, test and evaluate our results and thinking with the wider team, to determine the possible and likely interpretations we had made.

Another potential drawback was that our participants might make only favourable, but biased comments about CAPLE/researchers, or ‘what they wanted to hear.’ We proactively and repeatedly reassured participants that we wished hear about ‘difficult’ things, and that this would not disadvantage them, but offer us valuable insights. The 2017 ‘second’ exit interview with a researcher unknown to the participant was also done to specifically counter this possible drawback. We felt somewhat reassured that participants felt able to offer negative feedback because such comments were received from the outset of CAPLE, such as one participant who reported feeling briefly ‘badgered’ by workshop questions.

We have confidence in our findings for several reasons:

- an appropriately small sample size for our aim to find rich, in-depth qualitative data about staff experiences; a larger group would offer more modest opportunities to develop the required participant long term engagement for this
- a rich data set which allowed substantial triangulation between multiple sources and the ability to confirm ‘what was effective for what’
- researchers who offered a consensus of expert judgment, gained from teaching and participating in staff development over many decades
- researchers with considerable experience in gathering and interpreting qualitative data, and from staff in clinical settings
- similar results from 2016 and 2017 projects

Another possible drawback of this project was that we were also unable to quantitatively or comparatively measure levels of staff engagement, which instead were reported by participants and interpreted by the researchers. We countered this by employing researchers with extensive experience in gauging cognitive engagement of adult learners, and by triangulating data between participants and specific data sources from both studies.

We also need to stress that conclusions from CAPLE 2016 & 2017 are emergent and generated from two specific departments in one specific hospital with two researcher/facilitators, each embedded in their own specific context of practice. Other staff and workplaces might generate different responses and challenges. One example of this might be that a close-knit staff group might demand a lesser focus on teacher ‘respect,’ perhaps, but require more input in terms of facilitation skill.

### Ideas for further research

1. To clarify *how* each positive value enhanced participant engagement, and the effects of other values on engagement.
2. To explore further ways to avoid ‘targeting’ staff and further develop ideas of ‘legitimate’ topics for workplaces which are different to those described here.
3. To understand whether CAPLE helped staff engage because of its nature as an optional research project, rather than a ‘reactionary’ intervention.

## Abbreviations

AGB: Althea Gamble Blakey (researcher and primary author)
CAPLE: Creating A Positive Learning Environment – the intervention study which was administered in the hospital in question
KSH: Kelby Smith-Han (researcher and co-author)
LA: Lynley Anderson (Principle Investigator)
TI: Trainee Intern – final (6th) year medical student

## Footnotes

1 In some medical schools, the female gender now constitute significantly more than 50% of the total student intake
